# Effective Degradation of Free Gossypol in Defatted Cottonseed Meal by Bacterial Laccases: Performance and Toxicity Analysis

**DOI:** 10.3390/foods13040566

**Published:** 2024-02-13

**Authors:** Liangyu Zhang, Hao Zheng, Xingke Zhang, Xiaoxue Chen, Yanrong Liu, Yu Tang, Wei Zhang, Zhixiang Wang, Lihong Zhao, Yongpeng Guo

**Affiliations:** 1 College of Animal Science and Technology, Henan Agricultural University, Zhengzhou 450046, China; zhly0371@163.com (L.Z.); april262022@163.com (H.Z.); 18239791502@163.com (X.Z.); 2597172790@163.com (X.C.); weizhang@henau.edu.cn (W.Z.); wzxhau@henau.edu.cn (Z.W.); 2 College of Animal Science and Technology, China Agricultural University, Beijing 100193, China; 15110578158@163.com (Y.L.); m17801115235@163.com (Y.T.); zhaolihongcau@cau.edu.cn (L.Z.)

**Keywords:** free gossypol, laccase, degradation, cottonseed meal

## Abstract

Cottonseed meal (CSM) is the major by-product of the cottonseed oil extraction process with high protein content. However, the presence of free gossypol (FG) in CSM severely restricts its utilization in the food and animal feed industries. The development of a biological strategy for the effective removal of FG in CSM has become an urgent need. In this study, three bacterial laccases including CotA from *Bacillus licheniformis*, CueO from *Escherichia coli*, and LcLac from *Loigolactobacillus coryniformis* were heterologously expressed and investigated for their FG degradation ability. The results showed that CotA laccase displayed the highest FG-degrading capacity among the three laccases, achieving 100% FG degradation at 37 °C and pH 7.0 in 1 h without the addition of a redox mediator. Moreover, in vitro and in vivo studies confirmed that the hepatotoxicity of FG was effectively eliminated after oxidative degradation by CotA laccase. Furthermore, the addition of CotA laccase could achieve 87% to 98% FG degradation in defatted CSM within 2 h. In conclusion, CotA laccase can be developed as an effective biocatalyst for the detoxification of FG in CSM.

## 1. Introduction

The rising need for feed proteins, driven by the increasing global consumption of animal products, is a great challenge to fulfill given the limited availability of conventional feed protein sources including soybean meal, rapeseed meal, and fish meal. Cottonseed meal (CSM) is the major by-product of cottonseed after oil extraction, which contains about 40% protein [1]. The global cottonseed production is about 42 million metric tons per year, and approximately 45% of CSM remains after the removal of oil, lint, and hulls [2]. However, the direct utilization of CSM as protein ingredients for non-ruminants and young ruminants is severely hampered owing to the presence of a type of toxic polyphenol called gossypol. Moreover, CSM has promising applications as food products and functional food supplements if the gossypol content is reduced to a safe level [3,4]. Gossypol is predominantly present in the cotton (*Gossypium hirsutum*) pigment gland and occurs in free and bound form. Bound gossypol (BG) is formed via a covalent bond between the phenolic and carbonyl groups of gossypol and the epsilon amino groups of arginine and lysine. BG cannot be absorbed during digestion and is nontoxic, but it will decrease the bioavailability of the bound amino acids [5]. On the other hand, exposure to free gossypol (FG) adversely affects consumer health. Clinical signs of acute gossypol poisoning are similar among humans and animals and include respiratory distress, weakness, anorexia, and even death [6]. In males, dietary ingestion of FG can inhibit sperm production and motility and also increase the incidence of sperm abnormalities [7]. In females, FG exposure can disrupt the estrous cycle, impair oocyte maturation, and reduce embryo development [8,9]. Moreover, FG has been documented to induce hepatotoxicity, immunotoxicity, and thyroid toxicity [6].

Traditional approaches for removing FG in cottonseed by-products mainly include mechanical processing, gamma irradiation, solvent extraction, and chemical treatment with ferrous sulfate or calcium hydroxide [10]. Although these methods have achieved some success in FG detoxification, there are several shortcomings limiting their large-scale applications, such as high processing costs, reduced palatability and nutritional quality, and harsh reaction conditions. In recent years, several FG-degrading bacteria have been isolated, including *Aspergillus terreus* YJ01 [11], *Lactobacillus agilis* WWK129 [12], *Raoultella* sp. YL01 [13], and *Bacillus coagulans* S17 [14]. Solid-state fermentation by FG-degrading bacteria has been found to be a feasible process for detoxifying gossypol while improving the nutritional value of CSM [14,15]. The process of microbial fermentation usually takes several days to two weeks and may suffer from unwanted bacterial contamination. Until now, very little progress has been made towards the identification of microbial enzymes that can catalyze FG degradation. The generalist moth *Helicoverpa armigera* is an important pest on cotton plants and has evolved a strategy to detoxify gossypol. The carboxylesterase CarE and P450 cytochrome CYP9A12 are reported to participate in FG degradation by *H. armigera* [16,17].

Laccases are a class of multicopper oxidases capable of catalyzing the oxidation of a broad range of polyphenolic compounds [18]. Moreover, the substrate scope of laccases can be further broadened with the help of redox mediators, which act as electron shuttles between the enzyme and the desired substrate. Laccases are green biocatalysts as they use molecular oxygen as a co-substrate and only generate water as a by-product [19]. To date, laccases have been successfully applied for the degradation of a lot of food contaminants and anti-nutritional components, including mycotoxins [20], biogenic amines [21], and sinapic acid [22]. The polyphenolic structure of FG makes this compound a potential substrate for laccases. Wang et al. recently reported that commercial laccase from *Trametes versicolor* could catalyze the intramolecular annulation of the hydroxyl and aldehyde groups of FG [23]. In this work, we studied the FG-degrading capacity of bacterial laccases for the first time. Three bacterial laccases including CotA from *Bacillus licheniformis* ANSB821, CueO from *Escherichia coli* K12, and LcLac from *Loigolactobacillus coryniformis* HAU111 were heterologously expressed and characterized for their ability to directly oxidize FG or indirectly oxidize the compound in the presence of redox mediator. Moreover, in vitro and in vivo toxicity assessments of FG degradation products by CotA laccase were carried out. To promote the application of bacterial laccases as green biocatalysts for detoxifying FG, we evaluated the performance of CotA laccase-catalyzed FG removal in CSM.

## 2. Materials and Methods

### 2.1. Chemicals and Reagents

Gossypol standard was purchased from Shanghai Yuanye Bio-Technology Co., Ltd. (Shanghai, China). 2, 2′-Azino-bis-(3-ethylbenzothiazoline-6-sulphonic acid) (ABTS), 1-hydroxybenzotriazole (HBT), acetosyringone (AS), syringaldehyde (SA), and isopropyl-β-d-thiogalactopyranoside (IPTG) were purchased from Sigma Aldrich Co., Ltd. (Shanghai, China).

### 2.2. Expression and Purification of Laccases

Three bacterial laccases including CotA (NCBI Accession Number: QAX90317.1) from *Bacillus licheniformis* ANSB821, CueO (NCBI Accession Number: NP414665) from *Escherichia coli* K12, and LcLac (NCBI Accession Number: PP066064) from *Loigolactobacillus coryniformis* HAU111 were heterologously expressed in this study. *E. coli* Rosetta (DE3) harboring the expression vector pET-31b-CotA was constructed in our previous study [20]. The genomic DNA of *E. coli* K12 and *L. coryniformis* HAU111 were extracted using the Rapid Bacterial Genomic DNA Isolation Kit (B518225, Sangon Biotech Co., Ltd., Beijing, China). PCR was conducted to clone the coding genes of CueO and LcLac with primers listed in Table S1. The amplified products were digested with *Nde* I and *Xho* I, ligated into linearized pET31b (+), and transformed into the host *E. coli* Rosetta (DE3). For the expression of the recombinant laccases, a single colony containing the recombinant plasmid was inoculated in 5 mL of LB medium containing 100 μg mL^−1^ of ampicillin and cultured overnight at 37 °C with shaking at 180 rpm. Next, 300 mL of freshly prepared LB medium was inoculated with 3 mL of bacterial culture and then incubated at 37 °C and 180 rpm until the optical density (OD_600_) reached approximately 0.6. The expression of recombinant laccases was induced by adding 2 mM of CuSO_4_ and 0.1 mM of IPTG, followed by incubation at 16 °C and 120 rpm for 20 h. The purification of recombinant laccases was conducted using Ni^2+^ affinity chromatography according to the previously published protocol [18]. The purity and concentration of recombinant laccases were analyzed using the SDS-PAGE and Bradford methods, respectively. The enzymatic activity of laccases was measured spectrophotometrically at 37 °C using ABTS (ε420 = 36,000 M^−1^ cm^−1^) as the substrate in sodium citrate buffer (100 mM, pH 4.0). One unit of laccase activity was defined as the amount of enzyme required for oxidizing 1 μM of ABTS per minute.

### 2.3. Characterization of FG Degradation Capacity of Laccases

The ability of three bacterial laccases, CotA, CueO, and LcLac, to degrade FG was investigated. First, the FG degradation experiment was conducted in the absence of a redox mediator. FG (20 μg mL^−1^) was incubated with 1.0 U mL^−1^ of CotA, CueO, or LcLac in sodium phosphate buffer (100 mM, pH 7.0) at 37 °C for 1 h. The control was prepared without the addition of laccase. The FG degradation percentage was calculated with the following formula: Dp = (1 − Ce/C_C_) × 100%, where Dp was the FG degradation percentage; Ce and C_C_ were the FG concentrations in experimental and control groups, respectively. The degradation of FG by the laccase–mediator system was also studied. Four redox mediators including ABTS, HBT, AS, and SA were investigated. The FG-degrading experiment was carried out in sodium phosphate buffer (100 mM, pH 7.0) containing 1.0 U mL^−1^ of laccase, 20 μg mL^−1^ of FG, and 1 mM of redox mediator at 37 °C for 1 h.

The enzymatic properties of CotA laccase for directly oxidizing FG were further characterized. The time-dependent FG degradation by different amounts of CotA laccase was studied. FG (20 μg mL^−1^) was incubated with 0.1, 0.2, and 0.5 U mL^−1^ of CotA laccase in sodium phosphate buffer (100 mM, pH 7.0) at 37 °C for 10, 20, 30, and 40 min. To determine the pH effect on CotA laccase-catalyzed FG degradation, 0.5 U mL^−1^ of CotA laccase was incubated with 20 μg mL^−1^ of FG in different pH buffers (3.0–9.0) at 37 °C for 10 min. The temperature effect on FG degradation was determined by incubating 0.5 U mL^−1^ of CotA laccase with 20 μg mL^−1^ of FG in sodium phosphate buffer (100 mM, pH 7.0) at 22 to 52 °C for 10 min. The effect of metal ions on CotA laccase-catalyzed FG degradation was determined by pre-incubating 0.5 U mL^−1^ of CotA laccase with 10 mM of each metal ion in sodium phosphate buffer (100 mM, pH 7.0) at 37 °C for 10 min. FG (20 μg mL^−1^) was then added, and the reaction mixture was incubated at 37 °C for 10 min.

### 2.4. Quantification of FG by HPLC

The FG concentration was determined by high-performance liquid chromatography (HPLC, 1290 Infinity II, Agilent, Waldbronn, Germany) equipped with a C18 column (Agilent ZORBAX, 4.6 × 250 mm, 5 μm) and a UV detector set at 235 nm. The mobile phase consisted of 0.2% phosphoric acid in water and acetonitrile (15:85, v/v) at a flow rate of 1.0 mL min^−1^. The sample injection volume was 20 µL.

### 2.5. Molecular Docking Study

To elucidate the structural feature of CotA laccase for oxidizing FG, the interaction between the enzyme and substrate was analyzed through molecular docking using AutoDock 4.2. The 3D structure of CotA laccase was built with the homology modeling tool SWISS-MODEL based on the crystal structure of *Bacillus subtilis* 168 CotA laccase (PDB ID: 2WSD). The protein structure of CotA laccase was processed to remove water, add hydrogens, and compute Gasteiger charges and then exported in PDBQT format. The 3D structure of FG (Compounds CID 3503) was obtained from the PubChem database. Molecular docking simulation was carried out within 100 independent genetic algorithm (GA) runs, with 2.5 × 10^7^ being the number of evaluations for the Lamarckian GA method. The docking models were sorted according to the binding energy, and the complex with the best conformation was selected for visualization and analysis using PyMOL 2.1.

### 2.6. Cytotoxicity Assessment of FG Degradation Products

Human fetal hepatocyte cell line L-02 was obtained from Tongpai Biotechnology Co., Ltd. (Shanghai, China). Cells were cultured in RPMI 1640 medium supplemented with 10% fetal bovine serum (FBS), 100 U mL^−1^ of penicillin, and 100 U mL^−1^ of streptomycin at 37 °C in a humidified incubator. The cell viability of L-02 cells exposed to different concentrations of FG was first studied. L-02 cells were seeded in 96-well plates at a density of 1 × 10^4^ cells per well and then cultured for 24 h. Afterwards, the cells were treated with various concentrations of FG (2.5, 5, 10, and 20 μg mL^−1^) for 24 h. Then, the cell viability was tested by CCK-8 assay (C0038, Beyotime Inst Biotech, Shanghai, China) according to the kit instructions. The FG degradation products were prepared by incubating 2 mg mL^−1^ of FG with 100 U mL^−1^ of CotA laccase in sodium phosphate buffer (100 mM, pH 7.0) at 37 °C for 1 h. FG and FG degradation products were sterilized by filtration using a 0.22 μm syringe filter and diluted 1:100 in RPMI 1640 medium. The cell viability of L-02 cells exposed to FG and FG degradation products for 24 h was determined with CCK-8 assay. Moreover, the lactate dehydrogenase (LDH) activity, malondialdehyde (MDA) content, reactive oxygen species (ROS) level, cell apoptosis rate, and mitochondrial membrane potential (MMP) of L-02 cells were also measured. L-02 cells were seeded in 6-well plates at a density of 3 × 10^5^ cells per well and treated with FG and FG degradation products for 24 h. The LDH activity of the culture supernatant was detected with a commercially available kit (BC0685, Solarbio Technology Co., Ltd., Beijing, China). L-02 cells were harvested with 500 μL of PBS and sonicated on ice, and the MDA content in the cell homogenate was measured using a commercially available kit (BC0025, Solarbio Technology Co., Ltd., Beijing, China). The ROS level was determined by a dichlorodihydro-fluorescein diacetate (DCFH-DA) assay (BL714A, Biosharp, China). L-02 cells treated with FG and FG degradation product were incubated with 10 μM of DCFH-DA for 30 min at 37 °C, followed by three more rinses in PBS, and the fluorescence intensity was captured with a fluorescence microscope (Olympus, Tokyo, Japan) and quantified by ImageJ software 1.8.0. The level of intracellular ROS generation was calculated as a ratio of the fluorescence intensity of treated cells over the control. Cell apoptosis was assessed using an Annexin V-fluorescein isothiocyanate (FITC) Apoptosis Detection Kit (BMS500FI-100, eBioscience, San Diego, CA, USA). L-02 cells treated with FG and FG degradation product were double-stained by Annexin V-fluorescein isothiocyanate (FITC) and propidium iodide (PI) and then subjected to flow cytometric analysis. MMP was measured by using the JC-1 Assay Kit (C2006, Beyotime Inst Biotech, Shanghai, China). L-02 cells treated with FG and FG degradation product were incubated with 5 μg mL^−1^ of JC-1 staining solution at 37 °C for 30 min. The fluorescence intensity of the JC-1 aggregates/monomers (red fluorescence for JC-1 aggregates, green fluorescence for JC-1 monomers) was visualized by a fluorescence microscope (Olympus, Tokyo, Japan) and quantified by ImageJ software. The MMP value was calculated as a ratio of the fluorescence intensity of JC-1 aggregates over JC-1 monomers.

### 2.7. In Vivo Toxicity Assessment of FG Degradation Products

Thirty-six BALB/c mice (5 weeks old, male, weighing 18–20 g) were purchased from Vital River Laboratory Animal Technology Co., Ltd. (Beijing, China). The mice were kept in filter-top polycarbonate cages (2 mice per cage) with ad libitum access to feed and water, in a temperature-controlled (22 ± 2 °C) animal care room. After acclimation for one week, the mice were randomly divided into three groups and treated orally once a day for 4 weeks as follows: the CON group (mice were treated with 100 mM, pH 7.0 sodium phosphate buffer); the FG group (mice were treated with 10 mg/kg BW of FG in 100 mM, pH 7.0 sodium phosphate buffer); and the CotA + FG group (mice were treated with 10 mg/kg BW of CotA laccase-catalyzed FG degradation products in 100 mM, pH 7.0 sodium phosphate buffer). At the end of the treatment period, all mice were fasted overnight, and blood samples were taken from the orbital venous plexus. Serum was separated from the whole blood by centrifugation at 2000× *g* for 10 min, and stored at −80 °C for further analysis. Mice were sacrificed by cervical dislocation, and liver samples were dissected immediately and washed with ice-cold sterilized saline. Some liver samples were preserved in 4% paraformaldehyde for further histopathological analysis, and the others were stored at −80 °C. The liver samples preserved in 4% paraformaldehyde were dehydrated, embedded, sectioned, and stained with hematoxylin–eosin (HE). The levels of alanine aminotransferase (ALT), aspartate aminotransferase (AST), interleukin-1β (IL-1β), interleukin-2 (IL-2), and tumor necrosis factor-α (TNF-α) in serum as well as superoxide dismutase (SOD), glutathione peroxidase (GSH-Px), catalase (CAT), hydrogen peroxide (H_2_O_2_), and malondialdehyde (MDA) in liver homogenate were determined using commercially available assay kits (Nanjing Jiancheng Bioengineering Institute, Nanjing, China).

### 2.8. Application of Laccase to Degrade FG in Defatted Cottonseed Meal

Five defatted cottonseed meal (CSM) samples were purchased from the local market and crushed through a 60-mesh sieve. Approximately 10 g of CSM was blended with 50 mL of water containing 200 U CotA laccase in a 250 mL Erlenmeyer flask, and incubated at 37 °C and 180 rpm for 2 h. The control was prepared without the addition of CotA laccase. FG content in the samples was extracted by the addition of 50 mL of acetone, and incubated at 37 °C and 180 rpm in a rotary shaker for 1 h. The supernatant was collected, and the extraction process was repeated twice. The extraction was combined and subjected to FG quantification by HPLC analysis as described in Section 2.4.

### 2.9. Statistical Analysis

The data were subjected to a one-way analysis of variance (ANOVA), followed by Tukey’s Honestly Significant Difference (HSD) test. The threshold for statistical significance was *p* < 0.05. The results were presented as mean ± standard deviation (mean ± SD).

## 3. Results and Discussion

### 3.1. Expression of Three Bacterial Laccases and Investigation of their FG-Degrading Capacity

**Figure 1:**
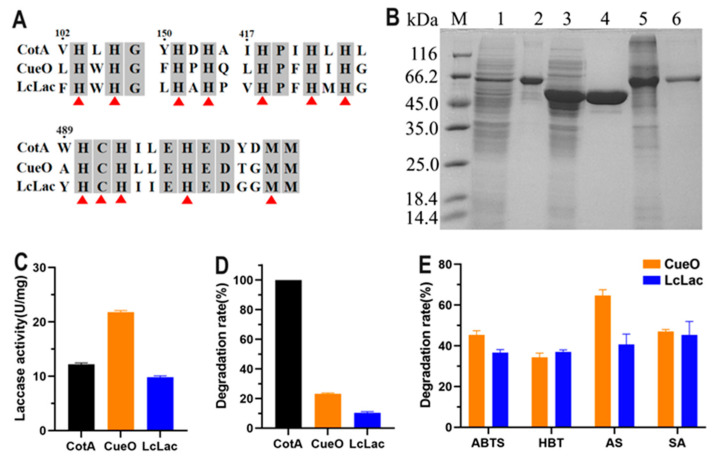
Expression of three bacterial laccases and investigation of their FG-degrading capacity. (**A**) Amino acid sequence alignment of the four copper-binding motifs. The twelve copper-binding residues are highlighted with a red arrow. (**B**) SDS-PAGE analysis of three bacterial laccases. Lane M, molecular mass standard; Lane 1, crude CotA extract; Lane 2, purified CotA; Lane 3, crude CueO extract; Lane 4, purified CueO; Lane 5, crude LcLac extract; and Lane 6, purified LcLac. (**C**) Laccase activity of CotA, CueO, and LcLac with ABTS as substrate. (**D**) Degradation of FG by CotA, CueO, and LcLac in the absence of redox mediator. Reaction condition: pH 7.0, 37 °C, initial FG content 20 μg mL^−1^, enzyme amount 1.0 U mL^−1^, and incubation time 1 h. (**E**) Degradation of FG by CueO and Lalac in the presence of redox mediator. Reaction condition: pH 7.0, 37 °C, initial FG content 20 μg mL^−1^, enzyme amount 1.0 U mL^−1^, redox mediator content 1 mM, and incubation time 1 h. All data are presented as mean ± SD (*n* = 3).

Three bacterial laccases, CotA from *B. licheniformis* ANSB821, CueO from *E. coli* K12, and LcLac from *L. coryniformis* HAU111 were expressed and investigated for their FG-degrading capacity in this study. CueO contained an N-terminal signal peptide as predicted by SignalP 5.0. The calculated molecular mass of CotA, the mature form of CueO and LcLac, was 59.1 kDa, 53.4 kDa, and 57.9 kDa. Sequence alignment showed that the three laccases shared less than 40% amino acid identity with each other. However, the four copper-binding motifs were highly conserved, which were composed of ten histidine residues, a cysteine residue, and a methionine residue (Figure 1A). The three laccases were fused with the 6 × His tag at the C-terminus and purified using Ni^2+^ affinity chromatography. As displayed in Figure 1B, the three purified recombinant laccases showed a clear single band on SDS-PAGE. The laccase activity of CotA, CueO, and LcLac was 12.2, 21.8, and 9.8 U mg^−1^ with ABTS as substrate (Figure 1C). The FG-degrading ability of the three laccases was initially evaluated without mediators. CotA laccase was found to be capable of catalyzing the complete degradation of FG, while CueO and LcLac only obtained a degradation rate of 23% and 10%, respectively (Figure 1D). Laccase catalysis can be enhanced by the inclusion of a redox mediator, which is first oxidized by the laccase and then the oxidized mediator catalyzes the oxidation of the target substrate [19]. The degradation of FG by CueO and LcLac in combination with a redox mediator was further investigated. As expected, the addition of redox mediators ABTS, HBT, AS, and SA could to some extent promote FG degradation by CueO and LcLac (Figure 1E). The CueO-AS system achieved the highest FG degradation rate of 65%. AS is a natural phenolic redox mediator that can be easily extracted from plant materials and by-products of the pulp manufacturing process [24]. The action mechanism of AS for substrate oxidation is based on hydrogen atom transfer (HAT) in which the mediator removes a hydrogen atom to generate phenoxyl radicals. Following this mechanism, AS has been used as a redox mediator to accelerate the laccase-catalyzed degradation of a wide range of recalcitrant aromatic compounds [25].

### 3.2. Enzymatic Characterization of CotA Laccase for Directly Oxidizing FG

**Figure 2:**
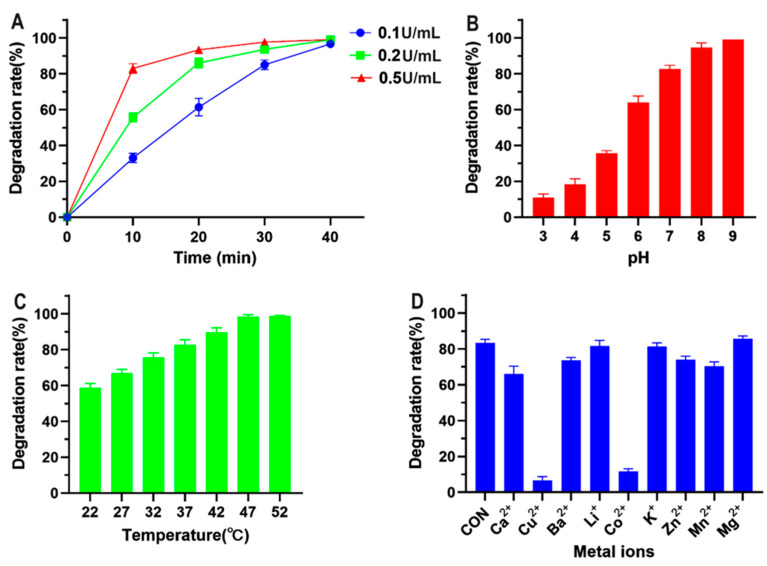
Enzymatic characterization of CotA laccase for directly oxidizing FG. (**A**) Time course of FG degradation by different amounts of CotA laccase. Reaction condition: pH 7.0, 37 °C, and initial FG content of 20 μg mL^−1^. (**B**) Effect of pH on CotA laccase-catalyzed FG degradation. Reaction condition: 37 °C, initial FG content of 20 μg mL^−1^, enzyme amount of 0.5 U mL^−1^, and incubation time of 10 min. (**C**) Effect of incubation temperature on CotA laccase-catalyzed FG degradation. Reaction condition: pH 7.0, initial FG content of 20 μg mL^−1^, enzyme amount of 0.5 U mL^−1^, and incubation time of 10 min. (**D**) Effect of metal ions on CotA laccase-catalyzed FG degradation. Reaction condition: pH 7.0, 37 °C, initial FG content of 20 μg mL^−1^, enzyme amount of 0.5 U mL^−1^, metal ion content of 10 mM, and incubation time of 10 min. All data are presented as mean ± SD (*n* = 3).

Taking into account that CotA laccase achieved the highest FG degradation rate in the absence of redox mediators among the three bacterial laccases, the enzymatic properties of CotA laccase for directly oxidizing FG were further investigated in this study. The time-dependent FG degradation by different amounts of CotA laccase is presented in Figure 2A. The degradation rate increased linearly from 33% at 10 min to 97% at 40 min at a CotA laccase dosage of 0.1 U mL^−1^. With the increase in enzyme amount to 0.2 and 0.5 U mL^−1^, the degradation rate within 10 min reached 56% and 83%, respectively. Moreover, more than 90% FG degradation was recorded at 30 and 10 min, respectively, by 0.2 and 0.5 U mL^−1^ CotA laccase. pH and temperature have important impacts on the biocatalytic capacity of enzymes. As shown in Figure 2B, the FG degradation rate increased when the pH was increased from 3.0 to 9.0, and FG was completely detoxified at pH 9.0. Similarly, the maximum activity of CotA laccase towards other phenolic substrates including zearalenone, 2,6-dimethoxyphenol, and syringaldazine was observed at neutral and alkaline pH conditions [20,26]. The influence of temperature on FG degradation by CotA laccase is presented in Figure 2C. The FG degradation rate increased from 59% to 98% with the increase in temperature from 22 to 47 °C, and the rate maintained a percentage of 99% at 52 °C. Indeed, CotA laccase orthologs from *Bacillus* species are well-known thermophilic enzymes with optimum temperatures ranging from 50 to 80 °C [27,28,29]. The impact of different metal ions on CotA laccase-catalyzed FG degradation is shown in Figure 2D. The presence of Li^+^, K^+^, and Mg^2+^ showed little effect on FG degradation by CotA laccase, whereas Ca^2+^, Ba^2+^, Zn^2+^, and Mn^2+^ at 10 mM resulted in a more than 10% reduction in the FG degradation rate. Moreover, Cu^2+^ and Co^2+^ could strongly inhibit the FG-oxidizing activity of CotA laccase. Consistently, Sun et al. [30] also recently documented the inhibitory effect of Cu^2+^ and Co^2+^ on CotA laccase for oxidizing alternariol, probably owing to their interactions with the electron transport system of the enzyme.

### 3.3. Molecular Docking of FG with CotA Laccase

**Figure 3:**
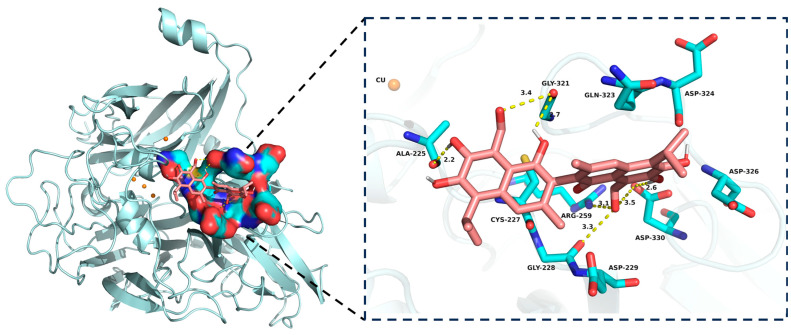
Molecular docking model of CotA laccase with FG. The left part shows the FG binding pocket of CotA laccase, and the right part displays the interaction between FG and amino acid residues of CotA laccase in detail. Hydrogen bonds are shown with yellow dashes with distances in angstroms.

Molecular docking was carried out to investigate the interaction model of FG with CotA laccase. As shown in Figure 3, FG was docked into the active pocket of CotA laccase with a binding free energy of −33.5 kJ/mol. The visualized image revealed that FG was producing only some fruitful binding conformations at the active site cavity due to the bulky planar ring structure of the substrate. Consistently, molecular docking studies revealed that ABTS did not occupy the whole part of the substrate pocket of CotA laccase [27]. The low binding free energy of the CotA/FG complex indicated that the configurations of FG within the active site were effective for productive binding. The substrate binding pocket was formed by Ala 225, Cys 227, Gly 228, Asp 229, Arg 259, Gly 321, Gln 323, Asp 324, Asp 326, and Asp 330. Hydrogen bonds played a key role in favoring the stability of the CotA/FG complex. There were seven hydrogen bonds formed between FG and amino acid residues Ala 225, Gly 228, Arg 259, Asp 330, and Gly 321. Among these amino acids, Gly 321 was also found to provide a hydrogen bond with aflatoxin B_1_ (AFB_1_) in a previous study [20]. The data of molecular docking were in agreement with the experimental results, both indicating that FG was a novel substrate of CotA laccase.

### 3.4. Cytotoxicity Assessment of FG Degradation Products

**Figure 4:**
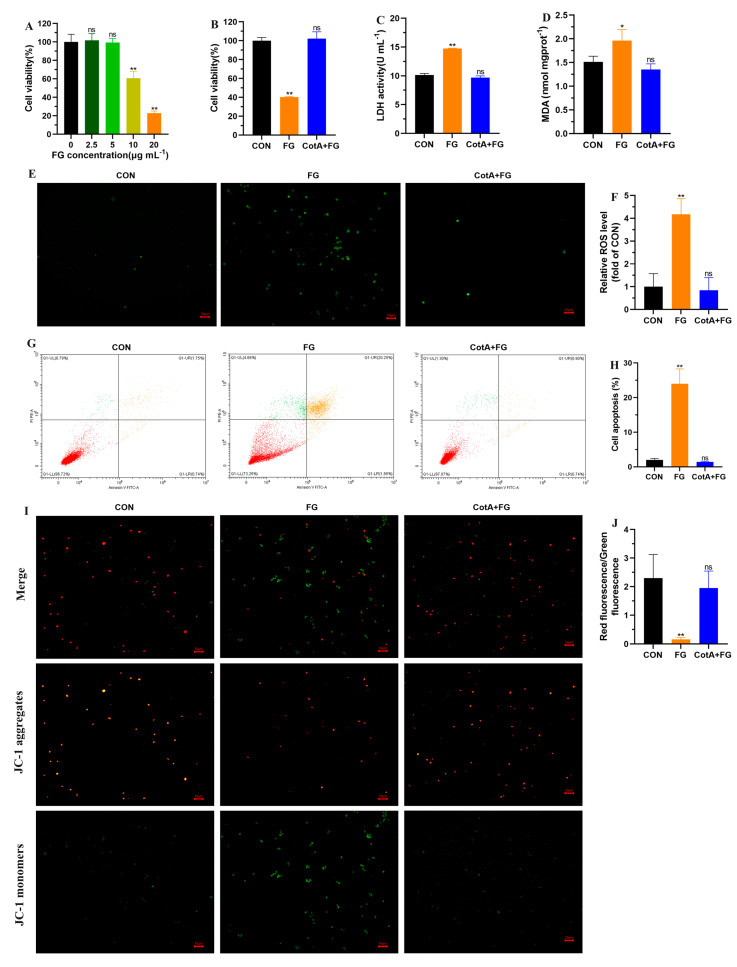
Cytotoxicity assessment of FG and its degradation products. (**A**) The viability of L−02 cells treated with different concentrations of FG for 24 h. (**B**) The viability of L−02 cells exposed to 20 μg mL^−1^ of FG and CotA laccase-catalyzed FG degradation products. For cell viability, values are represented as the mean ± SD (*n* = 6). (**C**) The leakage of LDH in the culture media. (**D**) Intracellular MDA content. (**E**,**F**) The fluorescence intensity of intracellular ROS. (**G**,**H**) Cell apoptosis rate measured by flow cytometry. Cells in the right lower and right upper quadrant were considered early and late apoptotic cells, respectively. (**I**,**J**) MMP analysis by JC−1 staining. Red fluorescence indicated the mitochondrial JC−1 aggregates and green fluorescence represented the JC−1 monomers. The MMP value was calculated as a ratio of the fluorescence intensity of JC−1 aggregates over JC−1 monomers. For C to J, data are presented as mean ± SD (*n* = 3). ns, not significant, * *p* < 0.05 and ** *p* < 0.01 in comparison with the CON group.

The liver, as the major site for xenobiotic metabolism and detoxification, is also the target organ affected by FG toxicity. Previous studies have documented that FG exposure caused hepatocyte degeneration, liver inflammation, and lipidosis in rats and chickens [31,32]. Thus, the cytotoxicity of FG degradation products was evaluated using L-02 hepatocytes in this study. The viability of L-02 cells decreased significantly after exposure to 10 and 20 μΜ of FG for 24 h (Figure 4A). However, pretreatment of 20 μΜ of FG with CotA laccase could alleviate the decrease in cell viability (Figure 4B). The damage of L-02 cells by FG was further confirmed by elevated LDH release in the culture supernatant, whereas no remarkable difference in LDH activity was observed between the control and CotA laccase-treated FG group (Figure 4C). Oxidative stress plays an important role in FG toxicity [8,33]. In this study, we found that FG exposure significantly increased intracellular MDA levels and led to ROS accumulation in L-02 cells (Figure 4D–F). Moreover, flow cytometry analysis indicated that oxidative stress triggered by FG exposure accelerated cell apoptosis (Figure 4G,H). Mitochondria are pivotal organelles mostly known as the “power plants” of the cells. Dysfunctional or damaged mitochondria can cause serious consequences, even resulting in cell death. MMP was determined using double fluorescence staining of mitochondria by JC-1, either as red fluorescent J-aggregates or as green fluorescent J-monomers. A significant increase in green fluorescence could be observed in L-02 cells incubated with FG, representing a decrease in MMP (Figure 4I,J), which was an important characteristic indicator of mitochondrial damage. Conversely, the FG degradation products by CotA laccase did not alter redox homeostasis in L-02 cells. These results suggested that the FG degradation products by CotA laccase were non-cytotoxic to L-02 cells.

### 3.5. In Vivo Toxicity Assessment of FG Degradation Products

**Figure 5:**
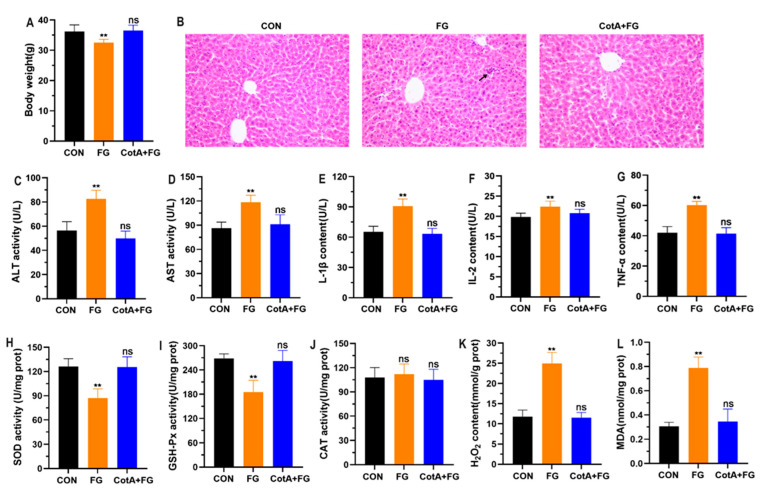
In vivo toxicity assessment of FG and its degradation products. (**A**) Body weight of mice at the end of the experiment. (**B**) Hematoxylin–eosin (HE) staining of representative liver sections. (**C**–**G**) Serum contents of ALT, AST, IL-1β, IL-2, and TNF-α. (**H**–**L**). Liver levels of SOD, GSH-Px, CAT, H_2_O_2_, and MDA. CON mice were treated with 100 mM, pH 7.0 sodium phosphate buffer; and FG mice were treated with 10 mg/kg BW of FG; CotA + FG mice were treated with 10 mg/kg BW of CotA laccase-catalyzed FG degradation products. All data are presented as mean ± SD (*n* = 6). ns, not significant, ** *p* < 0.01 in comparison with the CON group.

In vivo toxicity assessment was further conducted in mice by oral administration of FG and CotA laccase-catalyzed FG degradation products for four consecutive weeks. The body weight of mice receiving FG showed a significant reduction compared with the control, whereas oral administration of FG degradation products by CotA laccase did not reduce the body weight of mice (Figure 5A). Histopathological examination indicated that FG exposure caused inflammatory cell infiltration in the liver, but no obvious hepatic pathological changes were found in the control and CotA + FG groups (Figure 5B). In the study of El-Sharaky et al. [34], a two-week intraperitoneal injection of gossypol at 20 mg/kg BW led to moderate destruction and fatty degeneration of hepatocytes in rats. Serum ALT and AST levels were measured to examine liver function. In comparison with the control group, FG treatment notably increased serum ALT and AST activities (Figure 5C,D). Similarly, Fonseca et al. [31] also documented that FG exposure induced liver injury and promoted the release of ALT and AST into serum. Inflammation is commonly observed during acute and chronic liver damage. In the current study, serum contents of inflammatory cytokines IL-1β, IL-2, and TNF-α were remarkably increased in mice orally administered with FG (Figure 5E–G). Consistent with the cytotoxicity test, the in vivo study also suggested that oxidative stress was involved in FG-induced hepatotoxicity. As shown in Figure 5H–J, FG treatment significantly reduced the activities of liver antioxidant enzymes SOD and GSH-Px, while CAT activity was not altered. In addition, a remarkable increase in H_2_O_2_ and MDA contents in liver tissues was observed in the FG group compared with the control group. In contrast, oral administration of FG degradation products in mice did not significantly change serum levels of ALT, AST, IL-1β, IL-2, and TNF-α as well as liver levels of SOD, GSH-Px, CAT, H_2_O_2_, and MDA. Taken together, the results from in vitro and in vivo studies confirmed that the hepatotoxicity of FG was effectively eliminated after oxidative degradation by CotA laccase.

### 3.6. Application of CotA Laccase to Degrade FG in Defatted Cottonseed Meal

**Figure 6:**
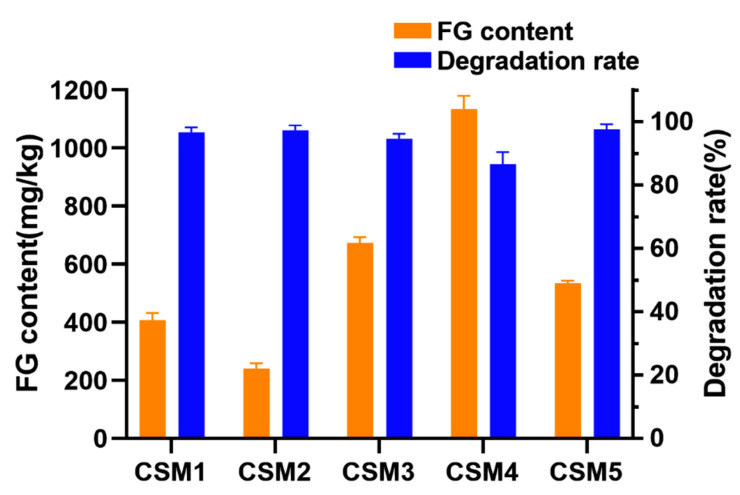
Degradation of FG in five defatted cottonseed meal samples (CSM1 to CSM5) by CotA laccase. FG content means the initial FG content in the samples. The degradation rate is calculated according to the residual FG content after CotA laccase treatment and the initial FG content in the samples. All data are presented as mean ± SD (*n* = 3).

The performance of CotA laccase for degrading FG in defatted CSM was investigated. As shown in Figure 6, the FG content in the five commercial CSM samples used in this study was 407, 240, 673, 1135, and 535 mg kg^−1^, and the FG degradation rate reached 97%, 97%, 95%, 87%, and 98%, respectively, after enzymatic treatment by CotA laccase for 2 h. In the previous study, only 70% of FG degradation in CSM at an initial content of 110 mg kg^−1^ was obtained by recombinant cytochrome P450 enzyme CYP9A12 after enzymatic treatment for 2.5 h [35]. The current study represents the first attempt to degrade FG in CSM with recombinant laccase. The FG detoxification rates achieved by CotA laccase were comparable to those obtained by microbial solid-state fermentation in previous studies. The FG content was decreased from 924 to 168 mg kg^−1^ with a degradation rate of 82% after fermentation with *Bacillus coagulans* S17 at 40 °C for 52 h [14]. Moreover, *Bacillus subtilis* M15 could reduce 93% of FG in CSM at an initial content of 784 mg kg^−1^ after anaerobic fermentation for 14 d [15]. In comparison with the solid-state fermentation method, laccase-catalyzed FG degradation could significantly shorten the incubation time. Thus, CotA laccase could be applied as a versatile biocatalyst to degrade FG in CSM.

## 4. Conclusions

In this work, three bacterial laccases, CotA, CueO, and LcLac, were expressed and investigated for their FG-degrading ability. CotA could effectively catalyze the direct oxidation of FG with a degradation rate of 100% at 37 °C and pH 7.0 in 1 h, whereas CueO and LcLac could only obtain FG degradation by 23% and 10% in the absence of a redox mediator. The addition of 1 mM of natural phenolic redox mediator AS could elevate FG degradation to 65% by CueO. The combined in vitro and in vivo studies revealed that CotA laccase-catalyzed FG degradation resulted in the elimination of its hepatotoxicity. Moreover, CotA laccase was found to be capable of degrading 87% to 98% of FG in defatted cottonseed meal within 2 h. Overall, this work provides an effective microbial enzyme agent for FG detoxification in cottonseed-derived products. Further study is still needed to elucidate the FG degradation products and mechanisms by CotA laccase.

## Data Availability

The data presented in this study are available on request from the corresponding author. The data are not publicly available due to privacy.

## Supplementary Materials

The following supporting information can be downloaded at: https://www.mdpi.com/article/10.3390/foods13040566/s1. Table S1: Primers for cloning of CueO and LcLac.
