# Supplementary material for: Effective Degradation of Free Gossypol in Defatted Cottonseed Meal by Bacterial Laccases: Performance and Toxicity Analysis
Source: Foods. 2024 Feb 13;13(4):566. doi: 10.3390/foods13040566 (PMC10888038; doi:10.3390/foods13040566)
Supplement: Supplementary file 1 [file foods-13-00566-s001.zip › foods-2852895-supplementary.pdf]

Table S1. Primers for cloning of CueO and LcLac.

Gene name	Primer
CueO	F: AATCATATGGCAGAACGCCCAACGTTAC<br>R: GGACTCGAGTACCGTAAACCCTAACATC
LcLac	F: GTGCATATGTCTGATAAGGTTTATACTG<br>R: CGACTCGAGCATCTTCATGCCCATTT
